# Stopping the effective non-fluoroquinolone antibiotics at day 7 vs continuing until day 14 in adults with acute pyelonephritis requiring hospitalization: A randomized non-inferiority trial

**DOI:** 10.1371/journal.pone.0197302

**Published:** 2018-05-16

**Authors:** Pavankumar Rudrabhatla, Surendran Deepanjali, Jharna Mandal, Rathinam Palamalai Swaminathan, Tamilarasu Kadhiravan

**Affiliations:** 1 Department of Medicine, Jawaharlal Institute of Postgraduate Medical Education and Research, Puducherry, India; 2 Department of Microbiology, Jawaharlal Institute of Postgraduate Medical Education and Research, Puducherry, India; Azienda Ospedaliera Universitaria di Perugia, ITALY

## Abstract

**Objective:**

To evaluate whether stopping the effective antibiotic treatment following clinical improvement at Day 7 (Truncated treatment) would be non-inferior to continued treatment until Day 14 (Continued treatment) in patients with acute pyelonephritis (APN) requiring hospitalization treated with non-fluoroquinolone (non-FQ) antibiotics.

**Methods:**

Hospitalized adult men and non-pregnant women with culture-confirmed APN were eligible for participation after they had clinically improved following empirical or culture-guided treatment with intravenous non-FQ antibiotic(s). We excluded patients with severe sepsis, abscesses, prostatitis, recurrent or catheter-associated urinary tract infection, or urinary tract obstruction. We randomized eligible patients on Day 7 of effective treatment and assessed them at Weeks 1 and 6 after treatment completion. The primary outcome was retreatment for recurrent urinary tract infection. The prespecified non-inferiority margin was 15%.

**Results:**

Between March 17, 2015 and August 22, 2016, we randomly allocated 54 patients—27 patients in each arm. Twenty-four (44%) patients were male, and 26 (48%) had diabetes mellitus. *Escherichia coli* was the most common urinary isolate (47 [87%] patients); 36 (78%) were resistant to ciprofloxacin. In all, 41 (76%) patients received amikacin-based treatment. At the end of 6 weeks, no patient in the truncated treatment arm required retreatment, whereas 1 patient in the continued treatment arm was retreated. Difference (90% CI) in retreatment was −3.7% (−15.01% to 6.15%). Upper bound of the difference (6.15%) was below the prespecified limit, establishing non-inferiority of truncated treatment. Asymptomatic bacteriuria at Week 6 was similar between the two arms (3/24 vs. 3/26; *P* = 1.0). Patients in the truncated treatment arm had significantly shorter hospital stay (8 [7–10] vs. 14 [14–15] days; *P* < 0.001) and less antibiotic consumption per patient (8.4 ± 2.8 vs. 17.4 ± 8.3 DDDs; *P* < 0.001).

**Conclusion:**

Stopping the effective non-FQ antibiotics following clinical improvement at Day 7 is non-inferior to continued treatment until Day 14 in selected patients with APN requiring hospitalization.

**Trial registration:**

Clinical Trials Registry-India; CTRI/2016/04/006810.

## Introduction

Acute pyelonephritis (APN) is one of the most common community-acquired infections requiring treatment with antibiotics. Traditionally, the duration of treatment for APN has been 10–14 days [1,2]. Based on recent trials, current clinical guidelines recommend shorter regimens of fluoroquinolones (FQs) for 5–7 days to treat uncomplicated APN in non-pregnant women in the outpatient setting [3–5]. However, in settings where the prevalence of FQ-resistance exceeds 10%, FQs are not preferred as first choice for treating APN [3]. Notably, in many settings including the Asia-Pacific region [6], several European and South American countries [7,8], and certain parts of the United States [9,10], prevalence of FQ-resistance among hospitalized patients with urinary tract infections considerably exceeds this threshold, necessitating treatment with alternative antibiotics such as third-generation cephalosporins, aminoglycosides, and beta-lactam/lactamase inhibitors [3,11–13].

While several clinical trials in the past had compared the clinical efficacy of these agents with another antibiotic such as FQs in patients with APN [14–16], none of the trials assessed the optimal duration of treatment regimens using non-FQ antibiotics other than co-trimoxazole; trials on hospitalized APN patients are particularly lacking [17]. Currently, the recommended treatment duration for non-FQ antibiotics is still 10–14 days [3,11–13], and hence the hospital stay is prolonged in the absence of suitable oral options and outpatient parenteral antibiotic treatment (OPAT) services. Notwithstanding, most APN patients treated with such antibiotics show clinical resolution within the first week of treatment [18], making clinicians contemplate whether these antibiotics could be stopped by the end of first week, without entailing an unduly high risk of retreatment for recurrent infection.

Given the higher chances of collateral ecological damage with the use of broad-spectrum antibiotics [19], it is important to know whether shorter treatment duration is good enough in hospitalized APN patients, which may often include men, post-menopausal women, and patients with diabetes mellitus. We therefore conducted a randomized controlled trial to test the hypothesis that, contingent upon clinical improvement, the effective non-FQ antibiotic regimen could be safely stopped at Day 7 while the risk of retreatment remains clinically acceptable in patients with APN requiring hospitalization.

## Materials and methods

We conducted a two-arm, parallel group, open-label, pragmatic randomized controlled trial with a non-inferiority design. This study was done at the Jawaharlal Institute of Postgraduate Medical Education and Research (JIPMER) hospital, Puducherry, India during the period March 17, 2015—August 22, 2016. Patients admitted with a provisional diagnosis of APN were eligible for this trial. The study protocol (S2 File) was reviewed and approved by the Institute Ethics Committee (Human studies) at JIPMER on January 6, 2015 (No. JIP/IEC/2014/8/381). We obtained informed written consent from all participants. This trial is registered on the Clinical Trials Registry-India (CTRI/2016/04/006810, URL: http://ctri.nic.in/Clinicaltrials/showallp.php?mid1=11429&EncHid=&userName=acute%20pyelonephritis). The protocol was submitted to the registry in March 2015 before the first patient was enrolled (Acknowledgment No. REF/2015/03/008632). However, due to a lapse in communication, the trial registration number was assigned only on April 8, 2016. The authors confirm that all ongoing and related trials for this drug/intervention are registered.

### Inclusion criteria

Patients admitted with a provisional diagnosis of APN were eligible for inclusion if they fulfilled all of the following criteria—i) age >18 years; ii) APN defined as fever (temperature ≥38 ^0^C recorded in hospital or a history of high grade fever) with dysuria and flank pain or costovertebral angle tenderness; iii) urine microscopy showing ≥10 pus cells/hpf or a positive dipstick leukocyte esterase test; and iv) pretreatment urine culture showing growth >10^5^ CFU/mL of pathogenic bacteria. In addition, eligible patients should have clinically improved following empirical or culture-guided antibiotic treatment and should be afebrile for >48 hours at the time of randomization (see below).

### Exclusion criteria

We excluded patients if they had any of the following—i) sterile or contaminated pretreatment urine culture; ii) recent urinary catheterization; iii) recurrent urinary tract infections in the past; iv) underlying obstructive uropathy; v) evidence of prostatitis/prostatic abscess (in men); vi) renal/perinephric collections or emphysematous changes in renal tissue; or vi) features of severe sepsis (septic shock requiring vasopressors, respiratory failure requiring mechanical ventilation, acute kidney injury requiring renal replacement therapy, or clinically manifest disseminated intravascular coagulation). We excluded pregnant and lactating women and patients on immunosuppressant drugs.

### Study procedure, treatment allocation, and interventions

All patients admitted to the medical wards during the study period with a diagnosis of APN were assessed for eligibility. The investigators had no role in the choice of empirical antibiotic(s), which was made by the treating physician. Due to a high prevalence of FQ-resistance in our setting [20], physicians seldom use FQs as the empirical choice for treating APN. A mid-stream urine sample was sent for culture before the first dose of antibiotic was administered. In all patients, an ultrasonographic assessment of kidneys, ureter, and bladder and prostate in men was done to exclude urinary tract obstruction and pus collections. One of the investigators (PR) followed the patients during hospital stay to assess resolution of fever and other symptoms and signs of APN. Pretreatment urine culture and susceptibility reports were available by Day 3. We excluded patients with sterile or contaminated pretreatment cultures. Patients who improved clinically within 72 hours of the empirical antibiotic regimen and whose pretreatment culture grew an organism susceptible to the given therapy continued with their treatment (referred to as the ‘empirical regimen’ patients); in them, urine culture was repeated on Day 4 of antibiotic treatment. In patients with suboptimal clinical response to empirical antibiotic and/or those with organisms resistant to the empirical choice on pretreatment culture, the antibiotic was revised by the treating physician (referred to as the ‘revised regimen’ patients). Once they clinically improved following treatment revision, urine culture was repeated on Day 4 of revised treatment. Patients with sustained clinical improvement on Day 7 of the effective antibiotic regimen (either empirical or revised) were eligible for randomization.

Since stratification of randomization by several variables is not effective in small-sized trials, we used a minimization method for randomization to balance the prognostic variables between the trial arms [21]. We considered the following factors for minimization—female gender, age ≥55 years, diabetes mellitus, peak serum creatinine ≥2 mg/dL, empirical or revised regimen, and aminoglycoside-based or non-aminoglycoside regimen. After obtaining informed written consent, one of the investigators not involved in enrollment (SD or TK) entered the details of consecutive patients into a computer program (MinimPy [22]) which assigned patients to either group in a ratio of 1:1. To maintain unpredictability (allocation concealment), we incorporated a biased-coin method with a base probability of 0.80. If the patient was allocated to truncated treatment (intervention arm), then the antibiotic treatment was stopped and he/she was discharged home. In those allocated to continued treatment (control arm), the same antibiotic regimen was continued until Day 14, and then they were discharged. Patients came back for follow-up visits at Week 1 and Week 6 after hospital discharge. All patients requiring retreatment during the follow-up period were to be retreated for 14 days irrespective of the trial arm they were allocated to. During the follow-up visits, they were enquired about any recurrent symptoms, and they provided a mid-stream urine sample for culture. Patients who did not turn up for scheduled visits were contacted over telephone and enquired about recurrence of symptoms, physician visits, and retreatment. The study methods are depicted in S1 Fig.

### Outcome measures

The primary outcome was retreatment for recurrent urinary tract infection during follow-up, up to 6 weeks after completion of antibiotic treatment. Treatment with antibiotics for any form of symptomatic urinary tract infection, which is inclusive of but not restricted to the clinical syndrome of APN, during the follow-up period was considered as retreatment. Protocol-specified secondary outcomes were duration of hospital stay (including re-admission, if any), antibiotic consumption per patient (expressed as defined daily doses [DDDs] [23], including retreatment if required), treatment-related side effects, and presence of asymptomatic bacteriuria at Week 1 and Week 6 after discharge.

### Sample size calculation

We chose a non-inferiority design for this study. We assumed that 5% of patients in the continued treatment arm would require retreatment. If there was truly no difference between continued and truncated treatments, then 27 patients were required in each arm to be 80% sure that the upper limit of a one-sided 95% confidence interval (or equivalently a 90% two-sided confidence interval) would exclude a difference in favor of continued treatment of more than 15% [24]. Allowing for a 15% loss to follow-up, the sample size was calculated as 31 patients in each arm. While the US Food and Drug Administration suggests a non-inferiority margin of 10% for trials on complicated urinary tract infections, it allows a higher margin in certain cases [25]. The Infectious Diseases Society of America had commented that a non-inferiority margin of 15% could be justified if there are critically important advantages such as shorter treatment duration and activity against drug-resistant uropathogens [26]. Hence, we set the non-inferiority margin *a priori* at 15%. This would translate roughly into a 33% reduction in antibiotic consumption (S2 Fig), which is a meaningful reduction from a stewardship perspective [27]. From a methodologic viewpoint, considering that the conservative estimate of the incremental efficacy of active treatments over placebo in clinical trials on complicated urinary tract infections is about 41% [28], a non-inferiority margin of 15% would still retain about 63% (relative) of active treatment effect, making it safe to conclude that the intervention is superior to putative placebo.

### Statistical analysis

We used a statistical software package for analysis (Stata/IC 12.1 for Windows, StataCorp LP, College Station, Texas, USA). We summarized normally distributed continuous variables as mean ± SD and tested between-group comparisons by independent *t*-test. We presented continuous variables with a skewed distribution, such as hospital stay, as median (IQR) and used Wilcoxon rank-sum test to test for significance. We summarized categorical variables as frequency with proportion (n [%]) and applied Fisher’s exact test for group comparisons. All tests were two-sided, and we considered *P* < 0.05 statistically significant.

We followed the effect estimation approach to assess non-inferiority for the primary outcome. We calculated confidence intervals (CIs) for the difference in retreatment proportions between the trial arms (Truncated treatment–Continued treatment) by Newcombe-Wilson hybrid score without applying a continuity correction [29]. Limits of the calculated 90% two-sided CIs correspond to respective one-sided 95% CIs (one-sided alpha of 5%). We concluded non-inferiority if the upper limit of the difference in retreatment did not exceed the prespecified non-inferiority margin of 15% in favor of the continued treatment arm. We used the Farrington-Manning exact test to calculate *P* value for non-inferiority (NCSS 11 Statistical Software, NCSS, LLC. Kaysville, Utah, USA). The primary analysis was by original assigned groups; however, any patient without primary outcome data was excluded. Since non-adherence to assigned treatment could bias the result toward non-inferiority, we also did a per protocol analysis for the primary outcome. Additionally, we performed a sensitivity analysis by the worst case scenario assuming that any patient lost to follow-up had the outcome of interest. In response to reviewer’s comments, we also examined non-inferiority at a one-sided alpha of 2.5% by using two-sided 95% CIs for the difference in retreatment for the primary and sensitivity analyses. We performed 6 *post hoc* subgroup analyses to explore whether the treatment effect differed across clinically meaningful subgroups. We applied a test of interaction (Mantel-Haenszel method) to assess whether the subgroup effects were different from the overall treatment effect.

## Results

**Fig 1:**
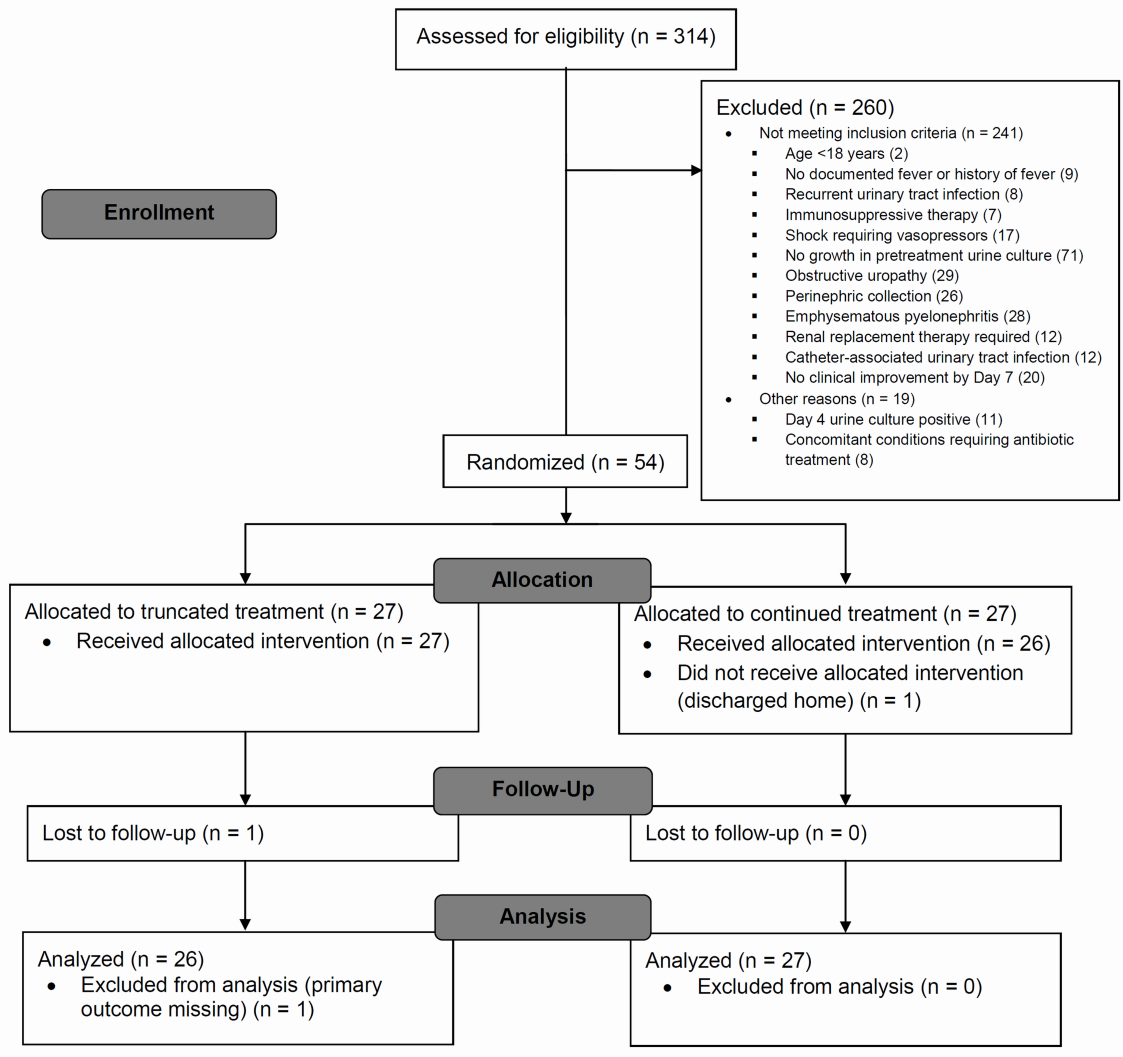
CONSORT flow diagram.

Between March 17, 2015 and August 22, 2016, we screened 314 patients admitted with a diagnosis of APN. After excluding 260 patients for reasons depicted in Fig 1, we randomly allocated 54 patients—27 patients to each trial arm. Since the accrual was slow and loss to follow-up was minimal, we stopped the trial once 54 patients were recruited. Follow-up of the last patient was completed on October 28, 2016. Overall, 24 (44%) patients were male; 26 (48%) patients had diabetes; and 17 (32%) patients had peak serum creatinine levels ≥2 mg/dL. *E*. *coli* was the most common pretreatment urinary isolate (47 [87%] patients) (Table 1). The other bacteria were *Enterococcus* spp. in 3, *Citrobacter koseri* in 2, and *Klebsiella pneumoniae* and *Pseudomonas* spp. in 1 each. Susceptibility pattern of *E*. *coli* isolates and details on the effective antibiotic regimen are presented in Table 1. An aminoglycoside (amikacin)-based regimen was used in 41 (76%) patients. Revision of initial antibiotic regimen was done in 12 (22%) patients. Of them, 11 were on single agent ceftriaxone, which was then revised to other regimens. The other patient was on meropenem initially, which was revised to amikacin.

**Table 1:** Characteristics of randomized patients at baseline.

Characteristic	Truncated treatment (n = 27)	Continued treatment (n = 27)
Age, years^a^	51 (42–60)	55 (42–60)
Age >55 years^b^	11 (41)	13 (48)
Female gender^b^	16 (59)	14 (52)
Diabetes mellitus^b^	13 (48)	13 (48)
**Clinical features**		
Fever	27 (100)	27 (100)
Dysuria	27 (100)	27 (100)
Frequency or urgency	8 (30)	9 (33)
Flank pain	21 (78)	21 (78)
Nausea/Vomiting	21 (78)	18 (67)
Pulse rate, per min^c^	102 ± 14	98 ± 15
Systolic blood pressure, mm Hg^c^	115 ± 17	119 ± 16
Diastolic blood pressure, mm Hg^c^	73 ± 9	76 ± 8
Renal angle tenderness	25 (93)	27 (100)
**Laboratory parameters**		
Serum creatinine, mg/dL^a^	1.4 (1.1–2.25)	1.3 (1.1–2.74)
Peak serum creatinine >2 mg/dL^b^	9 (33)	8 (30)
Total leukocyte count, per μL^c^	14577 ± 4444	14373 ± 4030
**Urinary pathogen isolated**		
*Escherichia coli*	24	23
*Enterococcus* spp.	1	2
*Citrobacter koseri*	1	1
*Pseudomonas* spp.	0	1
*Klebsiella pneumoniae*	1	0
**Antibiotic resistance among *E*. *coli* isolates (No. resistant / No. tested [%])**		
Ciprofloxacin	18/24 (74)	18/22 (82)
Ceftriaxone	19/24 (79)	17/23 (74)
Ceftazidime	17/23 (74)	17/23 (74)
Cefoperazone-sulbactam	0/7 (0)	1^d^/13 (8)
Meropenem	1^d^/22 (5)	0/22 (0)
Gentamicin	15/23 (65)	10/13 (77)
Amikacin	0/24 (0)	1^d^/23 (4)
Nitrofurantoin	0/6 (0)	0/13 (0)
**Effective antibiotic regimen**		
Ceftriaxone	5	5
Amikacin	12	10
Piperacillin-tazobactam	—	1
Cefoperazone-sulbactam	2	0
Ceftriaxone + amikacin	4	6
Piperacillin-tazobactam + amikacin	2	2
Cefoperazone-sulbactam + amikacin	2	2
Meropenem + amikacin	—	1
Aminoglycoside-based regimen^b^	20	21
Revised treatment regimen^b^	6	6

All data presented as n (%), unless indicated

^a^ = Data presented as median (IQR)

^b^ = Factors considered for minimization

^c^ = Data presented as mean ± SD

^d^ = Reported as intermediate susceptible.

Dosage of amikacin was 15 mg/kg q.d. in the presence of normal renal function, modified according to creatinine clearance otherwise; Dosage of ceftriaxone was 2 g q.d.; cefoperazone-sulbactam 2.0 g b.i.d. (n = 5), 1.5 g b.i.d. (n = 1); piperacillin-tazobactam 2.25 g q.i.d. (n = 4), 4.5 g q.i.d. (n = 1); and meropenem 1 g t.i.d. (n = 1).

All enrolled patients had clinical improvement with resolution of fever and urinary symptoms by Day 3 of effective antibiotic regimen. Day 4 urine cultures were sterile in 38 patients and were contaminated in 2 patients. In 3 patients, the Day 4 cultures grew an organism different from the pretreatment isolate, which was considered non-significant in view of the clinical improvement. Day 4 urine culture report was missing in 11 patients. However, all of them had sustained clinical improvement through Day 7 before randomization.

Baseline characteristics were well balanced between the two trial arms (Table 1). Of the 27 patients randomized to continued treatment, 1 patient was erroneously discharged on Day 7 and hence did not receive the assigned intervention. However, information on primary outcome was available for this patient and was retained in the primary analysis. Of the 27 patients randomized to truncated treatment, 1 patient was lost to follow-up after hospital discharge and was excluded from analysis for primary outcome (Fig 1). Two other patients did not turn up for any of the post-treatment assessments. However, they were contacted over telephone, and it was confirmed that they had no recurrent symptoms or retreatment.

**Fig 2:**
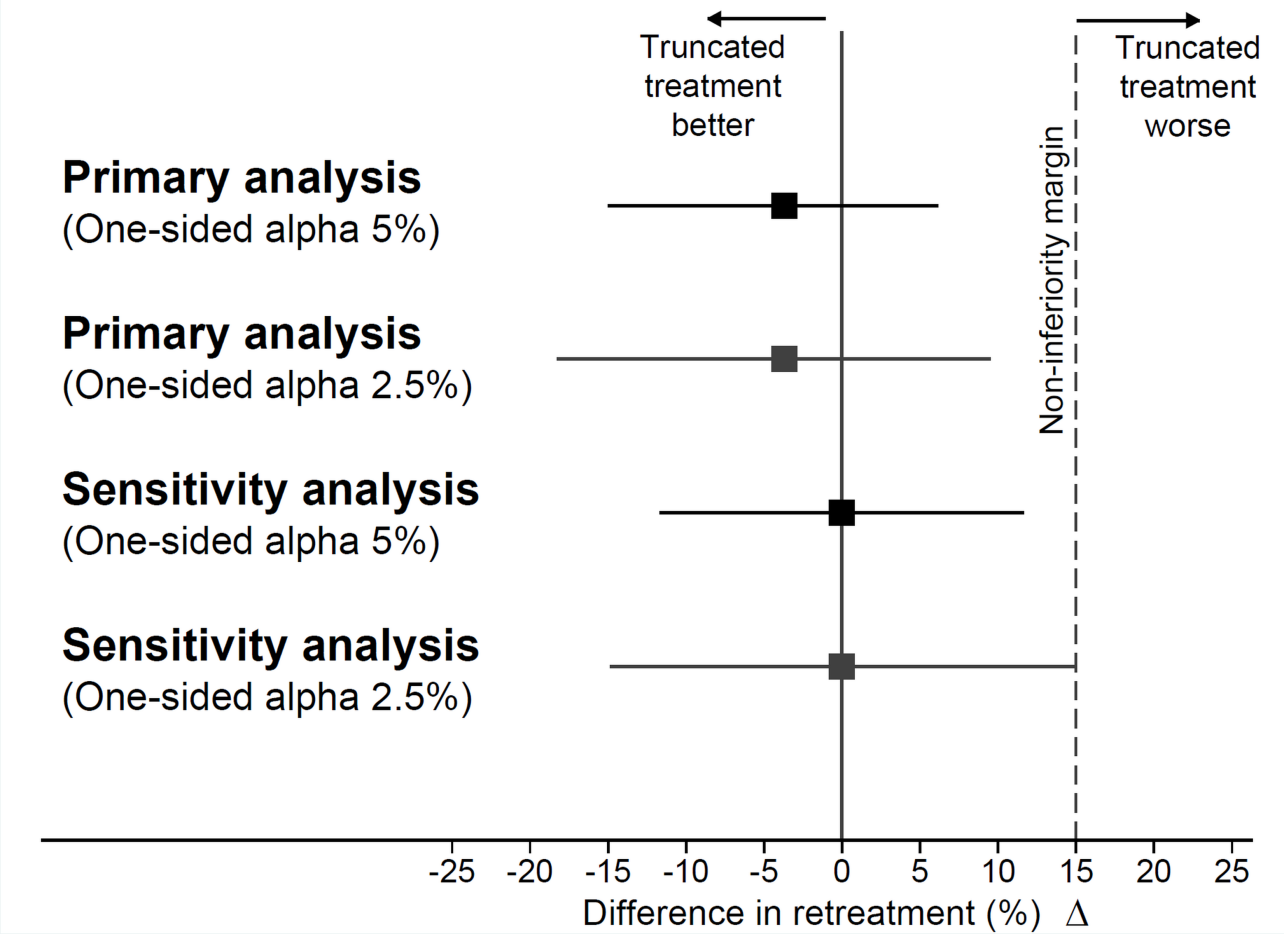
Non-inferiority assessment for the primary outcome. Point estimate of the difference in retreatment between trial arms (Truncated treatment–Continued treatment) is depicted by solid boxes. Error bars represent two-sided 90% CIs for one-sided alpha of 5% and two-sided 95% CIs for one-sided alpha of 2.5%, upper limits of which correspond to one-sided 95% and 97.5% CIs respectively.

**Fig 3:**
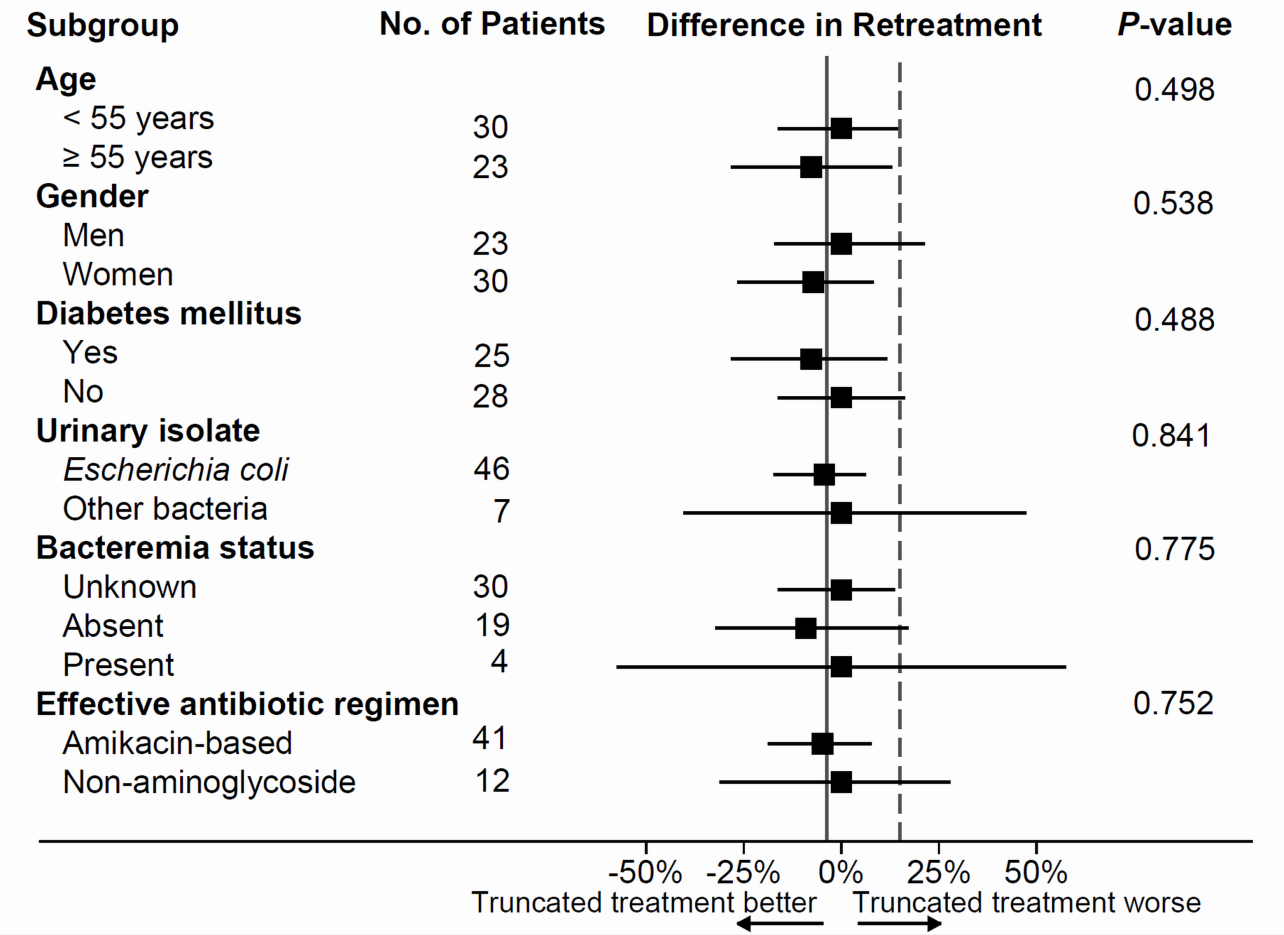
Subgroup analyses. Solid vertical line represents the overall treatment effect. Dotted line indicates the non-inferiority limit. Subgroup effects are presented as point estimates (solid boxes) with 90% CI. *P*-values are for a test of interaction.

During the 6 weeks follow-up period, no patient in the truncated treatment arm required retreatment, whereas 1 patient in the continued treatment arm was retreated for recurrent urinary tract infection. This was a lady with diabetes who had received amikacin-based regimen for 14 days. Four weeks after hospital discharge, she developed fever, dysuria, and lower abdominal pain; urine culture showed significant growth of *E*. *coli*. She was retreated with amikacin for 14 days. The difference (90% CI) in retreatment between the trial arms was −3.7% (−15.01% to 6.15%). Upper bound of the CI for the difference in retreatment in favor of the continued treatment arm (6.15%) was well below the prespecified margin of 15%, establishing non-inferiority of truncated treatment as compared to continued treatment (Fig 2). Non-inferiority criterion was met on a per protocol analysis also (difference in retreatment = −3.85% [−15.53% to 6.04%]). Non-inferiority of the truncated treatment arm was also robust to sensitivity analysis by the worst case assumption (1/27 vs. 1/27; Difference = 0% [-11.67% to 11.67%]). Truncated treatment remained non-inferior when re-examined at a one-sided alpha of 2.5% (primary analysis -3.7% [-18.28% to 9.52%]; sensitivity analysis 0% [95% CI -14.89% to 14.89%]; Fig 2). On *post hoc* subgroup analyses, there was no evidence to suggest that the treatment effect differed by characteristics such as age, gender, diabetes, urinary pathogen, presence of bacteremia, and use of aminoglycoside (Fig 3).

Patients randomized to truncated treatment had a significantly shorter hospital stay and less antibiotic consumption (Table 2). Mean antibiotic consumption in the truncated treatment arm was 48% lower as compared to the continued treatment arm. The mean (95% CI) difference in antibiotic consumption per patient was 9.01 (5.64 to 12.39) DDDs. When the analysis was restricted to patients with infections caused by ciprofloxacin-resistant organisms (n = 40), the difference between trial arms in antibiotic consumption (8.4 ± 3.2 vs 16.8 ± 8.5 DDDs; *P* < 0.001) and hospital stay (8 [7–10] vs 14 [14–14.5] days; *P* < 0.001) remained significant. Treatment-related side-effects were less frequent in the truncated treatment arm—1 patient each developed diarrhea and amikacin-related acute kidney injury. Whereas, in the continued treatment arm, 3 patients had thrombophlebitis, 2 patients had vomiting, 2 patients developed diarrhea, and 1 patient developed hypokalemia possibly related to piperacillin-tazobactam.

**Table 2:** Treatment outcomes.

Outcome measure	Truncated treatment (n = 27)	Continued treatment (n = 27)	*P*-value
**Primary outcome**			
Retreatment	0/26	1/27	0.009^a^
**Secondary outcomes**			
Antibiotic consumption per patient, DDDs^b^	8.4 ± 2.8	17.4 ± 8.3	<0.001
Hospital stay, days^c^	8 (7–10)	14 (14–15)	<0.001
*Urine culture—1 week after hospital discharge*			
No growth	18/22	19/23	
Asymptomatic bacteriuria	4/22	2/23	0.414^d^
Contaminated	—	2/23	
Not done	5/27	4/27	
*Urine culture—6 weeks after hospital discharge*			
No growth	19/24	19/26	
Asymptomatic bacteriuria	3/24	3/26	1.0^d^
Contaminated	1/24	3/26	
Yeast grown	1/24	1/26	
Not done	3/27	1/27	

DDD = Defined daily dose

^a^ = *P*-value is for non-inferiority

^b^ = Data presented as mean ± SD

^c^ = Data presented as median (IQR)

^d^ = *P*-value for presence of asymptomatic bacteriuria

A total of 9 patients did not come for the Week 1 follow-up. In the remainder (n = 45), the median time to first follow-up after discharge was 10 days (IQR 7–15 days). No patient reported persistent or recurrent urinary symptoms. There was no difference in the prevalence of asymptomatic bacteriuria between the two arms at Week 1 (Table 2). Fifty patients completed the second follow-up visit at Week 6, with a median visit time of 50 days (IQR 43–66 days). At Week 6 visit, a total of 6 patients had asymptomatic bacteriuria; 1 patient in the continued treatment arm reported mild dysuria, but no fever. Although she had a positive urine culture, she was considered not having a urinary tract infection and was not retreated. There was no significant difference in the presence of asymptomatic bacteriuria between the two arms at 6 weeks (Table 2; 3 in either arm). Four patients had asymptomatic bacteriuria at both Week 1 and Week 6–2 patients in each arm, and 3 of them had diabetes.

## Discussion

We found that stopping the effective non-FQ antibiotic treatment at Day 7, once sustained clinical improvement occurs, was not associated with an excess risk of retreatment as compared to continued treatment until Day 14 in selected hospitalized patients with APN. Our findings extend the available evidence base for shorter duration antibiotic treatment for APN to include non-FQ regimens and patient populations such as hospitalized patients. Understandably, this strategy was associated with a significant decrease in antibiotic consumption, length of hospital stay, and probably treatment-related side-effects. This trial was conducted in a setting with a high prevalence of uropathogens resistant to FQs and third-generation cephalosporins. Although we excluded patients with catheter-associated and recurrent urinary tract infections, severe sepsis, men with prostatic involvement, and patients who might require a surgical intervention in addition to antibiotic treatment such as those with emphysematous APN, renal/perinephric abscess, or obstructive uropathy, we tried to maintain a pragmatic approach by including patients with well-recognized risk factors for complicated APN, like male gender, diabetes, acute kidney injury, as well as failure of the empirical regimen. Moreover, this trial was not restricted to a particular organism or an antibiotic.

If one carefully looks at the reasons for exclusion, of the 260 patients with APN excluded from this trial, the findings of this trial could be reasonably extrapolated to those aged <18 years and those without documented fever or a positive baseline urine culture. Likewise, the trial findings could be cautiously applied to patients on immunosuppressive therapy and those with recurrent urinary infection, septic shock, or renal failure provided they show sustained clinical improvement by Day 7 of treatment. Clinical trials involving these subsets of patients to reliably inform the duration of antibiotic treatment individually in each of these small subsets are unlikely to be carried out in the future. On the other hand, the present findings cannot be extrapolated to patients with urinary obstruction, perinephric collections, emphysematous APN, persistent clinical symptoms by Day 7, or positive Day 4 urine cultures. Further clinical trials would be required to define the optimal duration of treatment in these subsets of patients. The present study had an open-label design. Lack of blinding could influence the decision to treat any recurrent symptoms with antibiotics. However, it would not influence the urine culture results. Moreover, both the patients with recurrent symptoms on follow-up were in the continued treatment arm. Thus, there was no reason to believe that a lack of blinding could have biased the results in favour of truncated treatment.

Resistance to amikacin was rare in the trial population, and most patients received amikacin-based treatment. Studies conducted in late 1980s and early 1990s had reported that aminoglycosides given for a shorter duration of 5 days resulted in bacteriological cure in more than 90% of patients with APN [30,31]. On the other hand, a systematic review of 26 randomized trials found that aminoglycosides were inferior to beta-lactams and FQs in achieving bacteriological cure at the end of treatment, but not at 30 days post-treatment in patients with urinary tract infections [32]. Thus, it is possible that shortening the treatment duration of aminoglycosides could result in an excess of retreatment for recurrent infection as compared to continued treatment for a longer duration. However, to our knowledge, this question has not been addressed by any of the clinical trials in the past.

Recently, in the face of extensive spread of antimicrobial resistance among pathogenic bacteria, shorter durations of antibiotic therapy have been studied and found to be effective for some common infections such as bacteremia and community-acquired pneumonia [33,34]. While there have been previous trials of shortened treatment duration in urinary tract infection, majority of patients included in such trials were otherwise healthy non-pregnant women [4,35]. Trials addressing treatment duration for APN in men as well as patients with co-morbidities are scarce [36]. Recently, a systematic review and meta-analysis of 8 randomized trials comparing efficacy of short (≤7 days) vs. longer antibiotic courses for treatment of APN concluded that 7 days of treatment is similar to longer treatment duration in terms of clinical and microbiological failure in patients with APN, including bacteremic patients [37]. However, all the 3 trials using non-FQ antibiotics (pivampicillin, pivmecillinam, cefixime) included in this meta-analysis were done before the year 2000. In the contemporary setting, where resistance to third-generation cephalopsorins is an important problem among hospitalized patients [9,38], ceftolozane/tazobactam and doripenem have been evaluated in randomized controlled trials for the treatment of APN [39,40]. However, the current evidence available for the use of non-FQs other than these drugs is largely from observational case series only [41–43]. The median treatment duration in these observational studies was 6–8 days, indicating that substantial number of patients were treated for more than 7 days duration. The present study provides evidence from a randomized controlled trial that the effective non-FQ antibiotic could be stopped at Day 7 once these patients have clinically improved.

One of the most significant studies on treatment duration in patients with febrile urinary tract infection has been recently reported from the Netherlands [44]. This study randomized patients to 7-days or 14-days of FQ-based treatment. Notably, men, post-menopausal women, patients with urogenital abnormalities, and co-morbidities were included in this trial. Overall, the clinical cure at Day 74–80 in the 7-days arm was non-inferior to the 14-days arm. However, short-term efficacy of the 7-days regimen in men at Day 10–18 days post-treatment was found to be inferior to the 14-days arm. The authors attributed this to clinically unapparent infection of prostatic tissue in men. Notwithstanding this finding, they proposed that shorter regimens for men might still be adequate since the need for additional antibiotic during follow-up was similar with both regimens, applying principles of antimicrobial stewardship. However, we did not did not find any difference between men and women during 6 weeks post-treatment with regard to retreatment or microbiological cure rates. Although the present study was not powered to address non-inferiority in individual subgroups such as men, it needs to be pointed out that “When evaluating a subgroup, the question is not whether the subgroup shows a statistically significant result but whether the subgroup treatment effects are significantly different from each other” [45]. While the only patient that required retreatment in our trial was female, asymptomatic bacteriuria during follow-up was equally seen among men and women. In our study, only 4 of the 24 men were treated with single agent amikacin, while the rest were treated with a cephalosporin (n = 17), piperacillin-tazobactam (n = 2), or meropenem (n = 1). While amikacin may not be effective in the acidic milieu of prostatic tissue, the latter drugs have been found to achieve therapeutic concentrations in the prostatic tissue [46,47].

The event rates in the present trial were quite low. Apart from the fact that as such recurrence following treatment for APN is uncommon [48], our trial eligibility criteria probably excluded patients at a higher risk for recurrent urinary tract infection. Another reason for the low event rates in our trial could be that we randomized only those patients with a sustained clinical improvement. For the same reason, we did not use ‘clinical cure’ as an outcome measure, which has been used in previous trials [35,44]. One notable difference between the present and previous trials is that we randomized patients on Day 7, for the reason that a clinician is at crossroads on Day 7, not at treatment initiation. Despite the fact that no events were observed in the 7-days arm, using a statistical rule-of-thumb [49], the upper limit of the 95% CI for retreatment in the truncated treatment arm would turn out to be 11.5% which is still under the prespecified non-inferiority limit.

One of the key measures adopted in any antibiotic stewardship program is intravenous to oral conversion of antibiotics [50]. In this study, all patients were given antibiotics intravenously throughout. Rates of resistance to FQs and third-generation cephalosporins in this trial were quite high and were similar to earlier published reports [51]. Many parts of world with widespread antimicrobial resistance are facing this challenge of scarcity of oral agents for treating community-acquired infections. In the absence of suitable oral options, availability of dedicated OPAT services could shorten the hospital stay in these patients. However, OPAT services are not widely available in most Asian countries including India [52]. At least in some of our trial participants who had infections caused by ciprofloxacin-susceptible organisms, the treatment could have been switched to oral FQs. However, for unclear reasons, the treating clinicians preferred to continue with the non-FQ regimens. Definitely, this highlights the need for a good stewardship program. Nonetheless, failure to switch to oral FQs in such patients would not affect our conclusion about the comparative efficacy of truncated treatment.

The strength of the present trial is its pragmatic outlook. This trial was conducted in real-life clinical settings, and the antibiotic choices were not controlled for the purpose of the study. Hence, the results are relevant to similar clinical settings where agents like third-generation cephalosporins, beta-lactam/beta-lactamase inhibitors, aminoglycosides as well as carbapenems are being used as first-line agents for the treatment of APN. The possible limitations of the present trial are—i) our findings may not be directly applicable to patients with severe complicated pyelonephritis, especially those with obstructive uropathy and other structural complications; ii) we did not perform strain typing of *E*.*coli* isolates to see whether the presence of bacteriuria after treatment in some patients indicates persistence of same strain or re-colonization by newer ones; and iii) this trial cannot inform the management of patients that remain urine culture positive on Day 4 of treatment despite clinical improvement. Further research is warranted to answer these pertinent clinical questions.

## Conclusions

We found that truncating the effective non-FQ antibiotic treatment at Day 7 is good enough in hospitalized APN patients without features of severe urosepsis and underlying urogenital tract abnormalities. Such a strategy could substantially cut down antibiotic consumption and shorten hospital stay in these patients.

## Supporting information

S1 Fig Schematic depiction of study procedure and follow-up. (PPTX)

S2 Fig Anticipated decrease in antibiotic consumption at a non-inferiority margin of 15%. Number of patients in each arm was assumed as 100, merely for ease of calculation. DDD = defined daily dose; ^a^ = Expected to be similar between the two arms since the randomization was minimized on this variable; ^b^ = Protocol-specified duration of retreatment was 14 days for both arms.
(PPTX)

S1 File CONSORT checklist. (DOC)

S2 File Study protocol. (DOCX)

S3 File De-identified dataset. (XLS)

## References

[1] Warren JW, Abrutyn E, Hebel JR, Johnson JR, Schaeffer AJ, Stamm WE. Guidelines for antimicrobial treatment of uncomplicated acute bacterial cystitis and acute pyelonephritis in women. Infectious Diseases Society of America (IDSA). Clin Infect Dis. 1999;29: 745–758. 10589881 10.1086/520427

[2] American College of Obstetricians and Gynecologists. ACOG Practice Bulletin No. 91: Treatment of urinary tract infections in nonpregnant women. Obstet Gynecol. 2008;111: 785–794. doi: 10.1097/AOG.0b013e318169f6ef 18310389 10.1097/AOG.0b013e318169f6ef

[3] Gupta K, Hooton TM, Naber KG, Wullt B, Colgan R, Miller LG, et al; Infectious Diseases Society of America; European Society for Microbiology and Infectious Diseases. International clinical practice guidelines for the treatment of acute uncomplicated cystitis and pyelonephritis in women: A 2010 update by the Infectious Diseases Society of America and the European Society for Microbiology and Infectious Diseases. Clin Infect Dis. 2011;52: e103–20. doi: 10.1093/cid/ciq257 21292654 10.1093/cid/ciq257

[4] Talan DA, Stamm WE, Hooton TM, Moran GJ, Burke T, Iravani A, et al Comparison of ciprofloxacin (7 days) and trimethoprim-sulfamethoxazole (14 days) for acute uncomplicated pyelonephritis pyelonephritis in women: a randomized trial. JAMA. 2000;283: 1583–1590. 10735395 10.1001/jama.283.12.1583

[5] Peterson J, Kaul S, Khashab M, Fisher AC, Kahn JB. A double-blind, randomized comparison of levofloxacin 750 mg once-daily for five days with ciprofloxacin 400/500 mg twice-daily for 10 days for the treatment of complicated urinary tract infections and acute pyelonephritis. Urology. 2008;71: 17–22. doi: 10.1016/j.urology.2007.09.002 18242357 10.1016/j.urology.2007.09.002

[6] Jean SS, Coombs G, Ling T, Balaji V, Rodrigues C, Mikamo H, et al Epidemiology and antimicrobial susceptibility profiles of pathogens causing urinary tract infections in the Asia-Pacific region: Results from the Study for Monitoring Antimicrobial Resistance Trends (SMART), 2010–2013. Int J Antimicrob Agents. 2016;47: 328–334. doi: 10.1016/j.ijantimicag.2016.01.008 27005459 10.1016/j.ijantimicag.2016.01.008

[7] Kahlmeter G, Åhman J, Matuschek E. Antimicrobial Resistance of Escherichia coli Causing Uncomplicated Urinary Tract Infections: A European Update for 2014 and Comparison with 2000 and 2008. Infect Dis Ther. 2015;4: 417–423. doi: 10.1007/s40121-015-0095-5 26507395 10.1007/s40121-015-0095-5 PMC4675763

[8] Bouchillon S, Hoban DJ, Badal R, Hawser S. Fluoroquinolone resistance among gram-negative urinary tract pathogens: global smart program results, 2009–2010. Open Microbiol J. 2012;6: 74–78. doi: 10.2174/1874285801206010074 23002406 10.2174/1874285801206010074 PMC3447161

[9] Talan DA, Takhar SS, Krishnadasan A, Abrahamian FM, Mower WR, Moran GJ; EMERGEncy ID Net Study Group. Fluoroquinolone-resistant and extended-spectrum β-lactamase–producing Escherichia coli infections in patients with pyelonephritis, United States. Emerg Infect Dis. 2016; 22: 1594–1603. 10.3201/eid2209.160148 PMC4994338 27532362

[10] Morrill HJ, Morton JB, Caffrey AR, Jiang L, Dosa D, Mermel LA, et al Antimicrobial resistance of Escherichia coli urinary isolates in the Veterans Affairs health care system. Antimicrob Agents Chemother. 2017;61: e02236–16. doi: 10.1128/AAC.02236-16 28193660 10.1128/AAC.02236-16 PMC5404570

[11] Hsueh PR, Hoban DJ, Carmeli Y, Chen SY, Desikan S, Alejandria M, et al Consensus review of the epidemiology and appropriate antimicrobial therapy of complicated urinary tract infections in Asia-Pacific region. J Infect. 2011;63: 114–123. doi: 10.1016/j.jinf.2011.05.015 21669223 10.1016/j.jinf.2011.05.015

[12] National Centre for Disease Control. Directorate General of Health Services. Ministry of Health & Family Welfare. Government of India. National Treatment Guidelines for Antimicrobial Use in Infectious Diseases Version 1.0; 2016 [cited 2018 February 17]. Available from: http://pbhealth.gov.in/AMR_guideline7001495889.pdf.

[13] Indian Council of Medical Research, Department of Health Research New Delhi, India. Treatment Guidelines for Antimicrobial Use in Common Syndromes; 2017 [cited 2018 February 17] Available from: http://www.icmr.nic.in/guidelines/treatment%20guidelines%20for%20antimicrobial.pdf

[14] Sandberg T, Englund G, Lincoln K, Nilsson LG. Randomised double-blind study of norfloxacin and cefadroxil in the treatment of acute pyelonephritis. Eur J Clin Microbiol Infect Dis. 1990;9: 317–323. 2197091 10.1007/BF01973737

[15] Melekos MD, Skoutelis A, Chryssanthopoulos C, Bassaris HP. A comparative study on aztreonam, ceftazidime and amikacin in the treatment of complicated urinary tract infections. J Chemother. 1991;3: 376–382. 1819621 10.1080/1120009x.1991.11739124

[16] Timmerman C, Hoepelman I, de Hond J, Boon T, Schreinemachers L, Mensink H, et al Open, randomized comparison of pefloxacin and cefotaxime in the treatment of complicated urinary tract infections. Infection. 1992;20: 34–37. 1563810 10.1007/BF01704892

[17] Johnson JR, Russo TA. Acute pyelonephritis in adults. N Engl J Med. 2018;378: 48–59. 29298155 10.1056/nejmcp1702758

[18] Johnson JR, Lyons MF 2nd, Pearce W, Gorman P, Roberts PL, White N, et al Therapy for women hospitalized with acute pyelonephritis: a randomized trial of ampicillin versus trimethoprim-sulfamethoxazole for 14 days. J Infect Dis. 1991;163: 325–330. 1988516 10.1093/infdis/163.2.325

[19] Meletiadis J, Turlej-Rogacka A, Lerner A, Adler A, Taconelli E, Mouton JW, the SATURN Diagnostic Study Group. Amplification of antimicrobial resistance in gut flora of patients treated with ceftriaxone. Antimicrob Agents Chemother. 2017;61: e00473–17. doi: 10.1128/AAC.00473-17 28807914 10.1128/AAC.00473-17 PMC5655041

[20] Mandal J, Acharya NS, Buddhapriya D, Parija SC. Antibiotic resistance pattern among common bacterial uropathogens with a special reference to ciprofloxacin resistant Escherichia coli. Indian J Med Res. 2012;136: 842–849. 23287133 PMC3573607

[21] Altman DG, Bland JM. Treatment allocation by minimisation. BMJ. 2005;330: 843 doi: 10.1136/bmj.330.7495.843 15817555 10.1136/bmj.330.7495.843 PMC556084

[22] Saghaei M, Saghaei S. Implementation of an open-source customizable minimization program for allocation of patients to parallel groups in clinical trials. J Biomed Sci Eng. 2011;4: 734–739.

[23] World Health Organization. Anatomical Therapeutic Chemical (ATC) Classification System: Guidelines for ATC Classification and DDD Assignment 2013: WHO Collaborating Centre for Drug Statistics Methodology; 2013 [cited 2018 February 17] Available from: https://www.whocc.no/filearchive/publications/1_2013guidelines.pdf

[24] Sealed Envelope Ltd. Power calculator for binary outcome non-inferiority trial; 2012 [cited 2018 February 17]. Available from: https://www.sealedenvelope.com/power/binary-noninferior/

[25] Food and Drug Administration, U.S. Department of Health and Human Services. Complicated Urinary Tract Infections: Developing Drugs for Treatment Guidance for Industry; 2015 [cited 2018 February 17]. Available from: https://www.fda.gov/downloads/Drugs/GuidanceComplianceRegulatoryInformation/Guidances/UCM070981.pdf

[26] Infectious Diseases Society of America. IDSA comments on FDA draft guidance for developing cUTI drugs; 2012 [cited 2018 February 17]. Available from: https://www.idsociety.org/uploadedFiles/IDSA/Policy_and_Advocacy/Current_Topics_and_Issues/Advancing_Product_Research_and_Development/Bad_Bugs_No_Drugs/Position_Papers/IDSA%20Comments%20on%20cUTI%20Draft%20Guidance%20052412.pdf

[27] Davey P, Brown E, Charani E, Fenelon L, Gould IM, Holmes A, et al Interventions to improve antibiotic prescribing practices for hospital inpatients. Cochrane Database Syst Rev. 2013;4: CD003543. 10.1002/14651858.CD003543.pub3 23633313

[28] Singh KP, Li G, Mitrani-Gold FS, Kurtinecz M, Wetherington J, Tomayko JF, et al Systematic review and meta-analysis of antimicrobial treatment effect estimation in complicated urinary tract infection. Antimicrob Agents Chemother. 2013;57: 5284–5290. doi: 10.1128/AAC.01257-13 23939900 10.1128/AAC.01257-13 PMC3811298

[29] Tandberg D. Improved confidence intervals for the difference between two proportions and number needed to treat (NNT). Version 1.49; 2014 [cited 2018 February 17]. Available from: www.cebm.net/wp-content/uploads/2014/04/Diff2PropCI49.xls

[30] Bailey RR, Peddie BA. Treatment of acute urinary tract infection in women. Ann Intern Med. 1987;107: 430. 10.7326/0003-4819-107-2-430_1 3619239

[31] Bailey RR, Lynn KL, Robson RA, Peddie BA, Smith A. Comparison of ciprofloxacin with netilmicin for the treatment of acute pyelonephritis. N Z Med J. 1992;105: 102–103. 1553112

[32] Vidal L, Gafter-Gvili A, Borok S, Fraser A, Leibovici L, Paul M. Efficacy and safety of aminoglycoside monotherapy: systematic review and meta-analysis of randomized controlled trials. J Antimicrob Chemother. 2007;60: 247–257. doi: 10.1093/jac/dkm193 17562680 10.1093/jac/dkm193

[33] Havey TC, Fowler RA, Daneman N. Duration of antibiotic therapy for bacteremia: a systematic review and meta-analysis. Crit Care. 2011;15: R267 doi: 10.1186/cc10545 22085732 10.1186/cc10545 PMC3388653

[34] Mandell LA, Wunderink RG, Anzueto A, Bartlett JG, Campbell GD, Dean NC, et al; Infectious Diseases Society of America; American Thoracic Society. Infectious Diseases Society of America/American Thoracic Society consensus guidelines on the management of community-acquired pneumonia in adults. Clin Infect Dis. 2007;44 Suppl 2: S27–S72. 17278083 10.1086/511159 PMC7107997

[35] Sandberg T, Skoog G, Hermansson AB, Kahlmeter G, Kuylenstierna N, Lannergård A, et al Ciprofloxacin for 7 days versus 14 days in women with acute pyelonephritis: a randomised, open-label and double-blind, placebo-controlled, non-inferiority trial. Lancet. 2012;380: 484–490. doi: 10.1016/S0140-6736(12)60608-4 22726802 10.1016/S0140-6736(12)60608-4

[36] van der Starre WE, van Dissel JT, van Nieuwkoop C. Treatment duration of febrile urinary tract infections. Curr Infect Dis Rep. 2011;13: 571–578. doi: 10.1007/s11908-011-0211-y 21882085 10.1007/s11908-011-0211-y PMC3207126

[37] Eliakim-Raz N, Yahav D, Paul M, Leibovici L. Duration of antibiotic treatment for acute pyelonephritis and septic urinary tract infection—7 days or less versus longer treatment: systematic review and meta-analysis of randomized controlled trials. J Antimicrob Chemother. 2013;68: 2183–2191. doi: 10.1093/jac/dkt177 23696620 10.1093/jac/dkt177

[38] Mazzariol A, Bazaj A, Cornaglia G. Multi-drug-resistant Gram-negative bacteria causing urinary tract infections: a review. J Chemother. 2017;29: 2–9. doi: 10.1080/1120009X.2017.1380395 29271736 10.1080/1120009X.2017.1380395

[39] Popejoy MW, Paterson DL, Cloutier D, Huntington JA, Miller B, Bliss CA, et al Efficacy of ceftolozane/tazobactam against urinary tract and intra-abdominal infections caused by ESBL-producing Escherichia coli and Klebsiella pneumoniae: a pooled analysis of Phase 3 clinical trials. J Antimicrob Chemother. 2017;72: 268–272. doi: 10.1093/jac/dkw374 27707990 10.1093/jac/dkw374

[40] Naber KG, Llorens L, Kaniga K, Kotey P, Hedrich D, Redman R. Intravenous doripenem at 500 milligrams versus levofloxacin at 250 milligrams, with an option to switch to oral therapy, for treatment of complicated lower urinary tract infection and pyelonephritis. Antimicrob Agents Chemother. 2009;53: 3782–3792. doi: 10.1128/AAC.00837-08 19581455 10.1128/AAC.00837-08 PMC2737884

[41] Park SH, Choi SM, Chang YK, Lee DG, Cho SY, Lee HJ, et al The efficacy of non-carbapenem antibiotics for the treatment of community-onset acute pyelonephritis due to extended-spectrum β-lactamase-producing Escherichia coli. J Antimicrob Chemother. 2014;69: 2848–2856. doi: 10.1093/jac/dku215 24928854 10.1093/jac/dku215

[42] Cho SY, Choi SM, Park SH, Lee DG, Choi JH, Yoo JH. Amikacin therapy for urinary tract infections caused by extended-spectrum β-lactamase producing Escherichia coli. Korean J Intern Med. 2016;31: 156–161. doi: 10.3904/kjim.2016.31.1.156 26767869 10.3904/kjim.2016.31.1.156 PMC4712420

[43] Polat M, Kara SS. Once-daily intramuscular amikacin for outpatient treatment of lower urinary tract infections caused by extended-spectrum β-lactamase-producing Escherichia coli in children. Infect Drug Resist. 2017;10: 393–399. doi: 10.2147/IDR.S148703 29138582 10.2147/IDR.S148703 PMC5674974

[44] van Nieuwkoop C, van der Starre WE, Stalenhoef JE, van Aartrijk AM, van der Reijden TJ, Vollaard AM, et al Treatment duration of febrile urinary tract infection: a pragmatic randomized, double-blind, placebo-controlled non-inferiority trial in men and women. BMC Med. 2017;15: 70 doi: 10.1186/s12916-017-0835-3 28366170 10.1186/s12916-017-0835-3 PMC5376681

[45] Moher D, Hopewell S, Schulz KF, Montori V, Gøtzsche PC, Devereaux PJ, et al CONSORT 2010 explanation and elaboration: updated guidelines for reporting parallel group randomised trials. BMJ. 2010;340: c869 doi: 10.1136/bmj.c869 20332511 10.1136/bmj.c869 PMC2844943

[46] Charalabopoulos K, Karachalios G, Baltogiannis D, Charalabopoulos A, Giannakopoulos X, Sofikitis N. Penetration of antimicrobial agents into the prostate. Chemotherapy. 2003;49: 269–279. doi: 10.1159/000074526 14671426 10.1159/000074526

[47] Lipsky BA, Byren I, Hoey CT. Treatment of bacterial prostatitis. Clin Infect Dis. 2010;50: 1641–1652. doi: 10.1086/652861 20459324 10.1086/652861

[48] Ki M, Park T, Choi B, Foxman B. The epidemiology of acute pyelonephritis in South Korea, 1997–1999. Am J Epidemiol. 2004;160: 985–993. doi: 10.1093/aje/kwh308 15522855 10.1093/aje/kwh308

[49] Hanley JA, Lippman-Hand A. If nothing goes wrong, is everything all right? Interpreting zero numerators. JAMA. 1983;249: 1743–1745. 6827763

[50] Barlam TF, Cosgrove SE, Abbo LM, MacDougall C, Schuetz AN, Septimus EJ, et al Implementing an Antibiotic Stewardship Program: Guidelines by the Infectious Diseases Society of America and the Society for Healthcare Epidemiology of America. Clin Infect Dis. 2016;62: e51–77. doi: 10.1093/cid/ciw118 27080992 10.1093/cid/ciw118 PMC5006285

[51] Center for Disease Dynamics, Economics & Policy. Antibiotic resistance of Escherichia coli in India. ResistanceMap, Washington DC; 2015 [cited 2018 February 17]. Available from: https://resistancemap.cddep.org/CountryPage.php?countryId=17&country=India

[52] Fisher D, Michaels J, Hase R, Zhang J, Kataria S, Sim B, et al Outpatient parenteral antibiotic therapy (OPAT) in Asia: missing an opportunity. J Antimicrob Chemother. 2017;72: 1221–1226. doi: 10.1093/jac/dkw551 28077673 10.1093/jac/dkw551

