# Supplementary material for: Stopping the effective non-fluoroquinolone antibiotics at day 7 vs continuing until day 14 in adults with acute pyelonephritis requiring hospitalization: A randomized non-inferiority trial
Source: PLoS One. 2018 May 16;13(5):e0197302. doi: 10.1371/journal.pone.0197302 (PMC5955556; doi:10.1371/journal.pone.0197302)
Supplement: S1 Fig — (PPTX) [file pone.0197302.s001.pptx]

## Slide 1
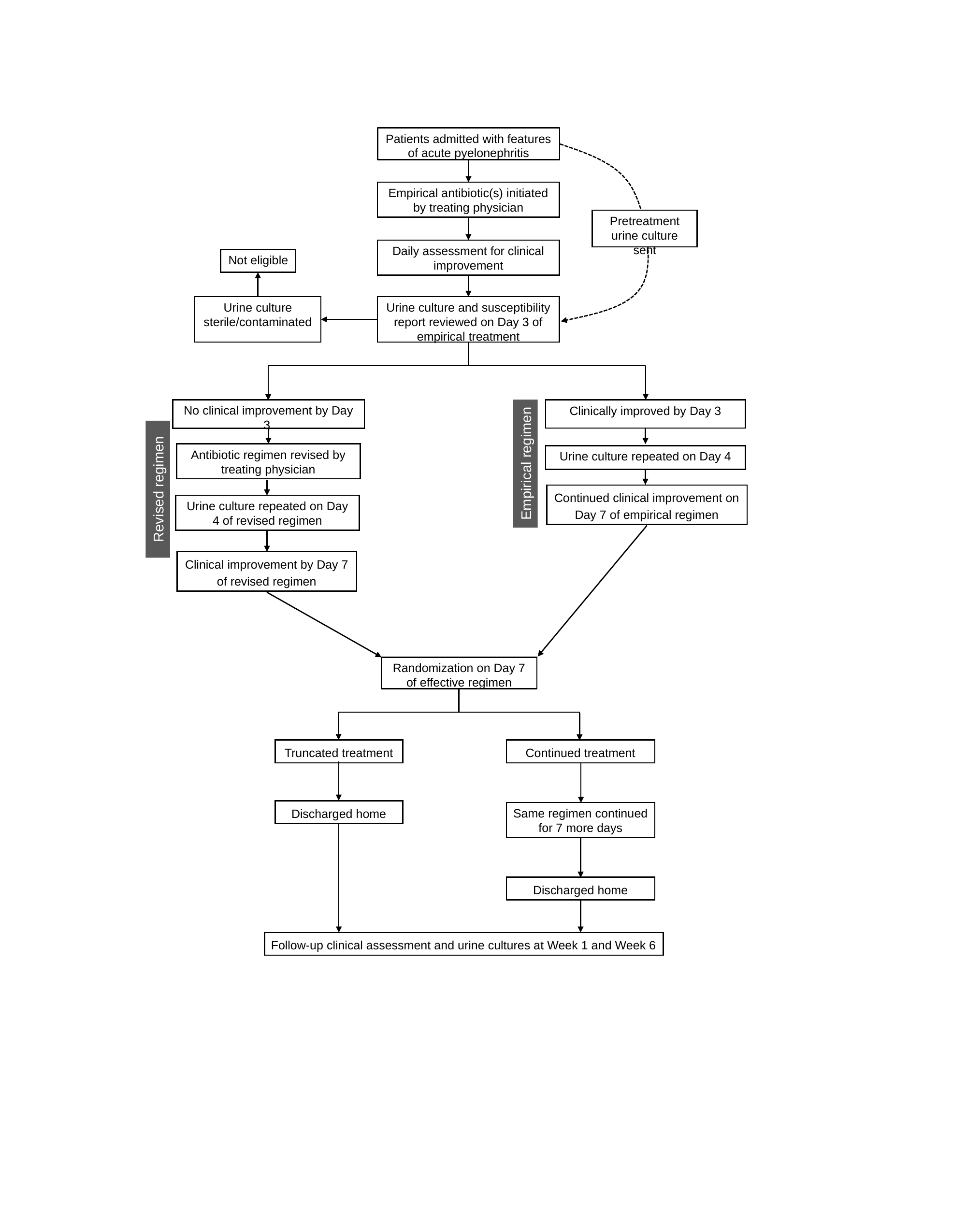

Patients admitted with features of acute pyelonephritis
Empirical antibiotic(s) initiated by treating physician
Pretreatment urine culture sent
Daily assessment for clinical improvement
Not eligible
Urine culture sterile/contaminated
Urine culture and susceptibility report reviewed on Day 3 of empirical treatment
No clinical improvement by Day 3
Clinically improved by Day 3
Antibiotic regimen revised by treating physician
Urine culture repeated on Day 4
Empirical regimen
Revised regimen
Continued clinical improvement on Day 7 of empirical regimen
Urine culture repeated on Day 4 of revised regimen
Clinical improvement by Day 7 of revised regimen
Randomization on Day 7
of effective regimen
Continued treatment
Truncated treatment
Discharged home
Same regimen continued
for 7 more days
Discharged home
Follow-up clinical assessment and urine cultures at Week 1 and Week 6
