# Supplementary material for: Stopping the effective non-fluoroquinolone antibiotics at day 7 vs continuing until day 14 in adults with acute pyelonephritis requiring hospitalization: A randomized non-inferiority trial
Source: PLoS One. 2018 May 16;13(5):e0197302. doi: 10.1371/journal.pone.0197302 (PMC5955556; doi:10.1371/journal.pone.0197302)
Supplement: S2 Fig — Number of patients in each arm was assumed as 100, merely for ease of calculation. DDD = defined daily dose; a = Expected to be similar between the two arms since the randomization was minimized on this variable; b = Protocol-specified duration of retreatment was 14 days for both arms. (PPTX) [file pone.0197302.s002.pptx]

## Slide 1
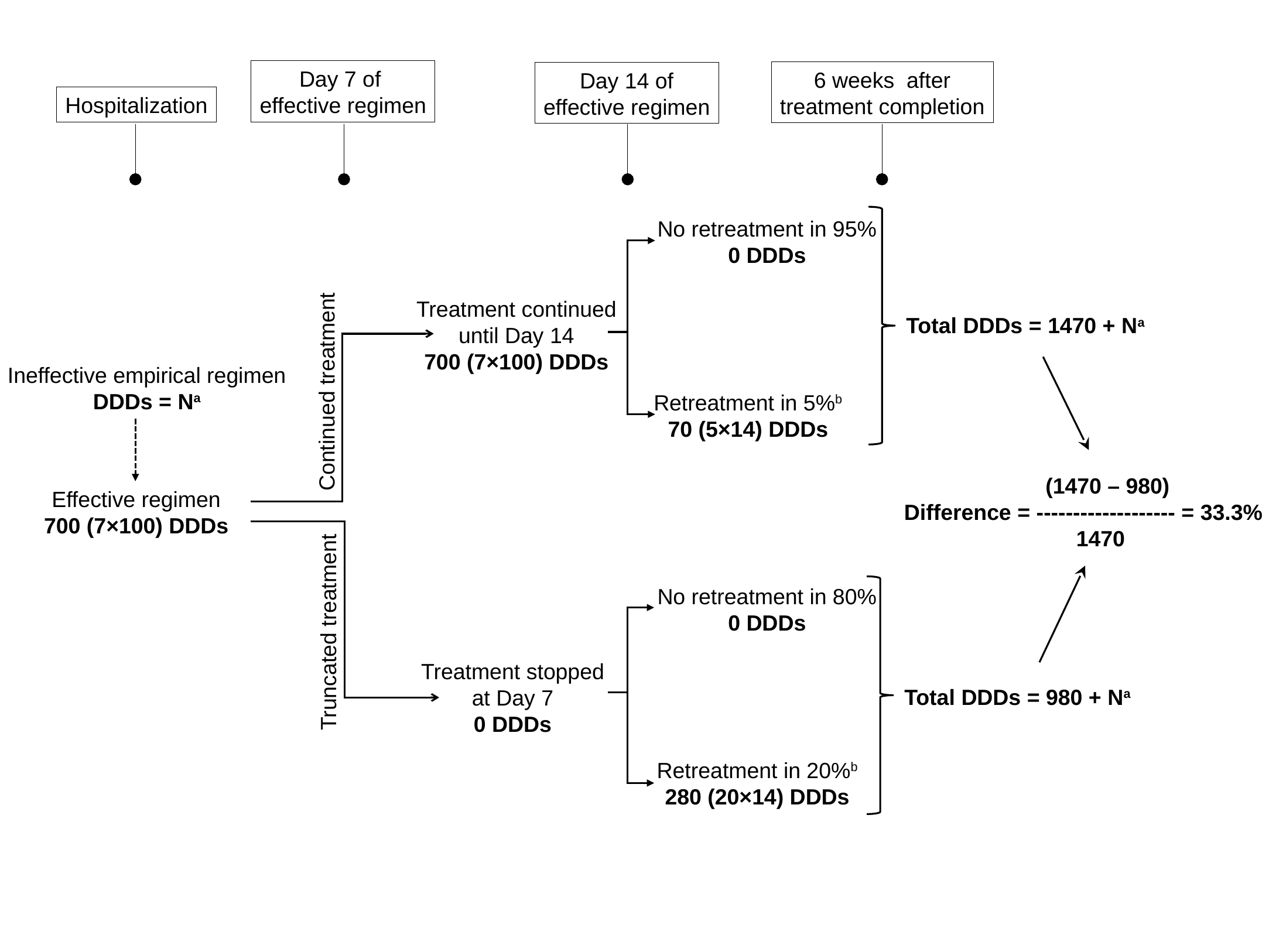

Day 7 of
effective regimen
6 weeks after
treatment completion
Day 14 of
effective regimen
Hospitalization
No retreatment in 95%
0 DDDs
Treatment continued
until Day 14
700 (7×100) DDDs
Total DDDs = 1470 + Na
Ineffective empirical regimen
DDDs = Na
Continued treatment
Retreatment in 5%b
70 (5×14) DDDs
 (1470 – 980)
Difference = ------------------- = 33.3%
 1470
Effective regimen
700 (7×100) DDDs
No retreatment in 80%
0 DDDs
Truncated treatment
Treatment stopped
at Day 7
0 DDDs
Total DDDs = 980 + Na
Retreatment in 20%b
280 (20×14) DDDs
