# Supplementary material for: Stopping the effective non-fluoroquinolone antibiotics at day 7 vs continuing until day 14 in adults with acute pyelonephritis requiring hospitalization: A randomized non-inferiority trial
Source: PLoS One. 2018 May 16;13(5):e0197302. doi: 10.1371/journal.pone.0197302 (PMC5955556; doi:10.1371/journal.pone.0197302)
Supplement: S2 File — (DOCX) [file pone.0197302.s004.docx]

**Final version of trial protocol approved by the Institute Ethics Committee**

**(Formatting changes have been done)**

**Form IA**

**Proforma to be submitted to the Institute Ethics Committee (Human Studies)**

(for projects other than those mentioned in Form I B)

***Kindly submit 15 copies of proforma and consent forms in 2 parts (in English and Tamil) to the Member Secretary, Institute Ethics Committee (Human Studies), JIPMER, Puducherry***

[Projects can be submitted throughout the year]

- 1. **Title of the project**: “Comparison of 7 days versus 14 days antibacterial treatment for hospitalized non-severe acute pyelonephritis patients – a randomized controlled trial”
  2. **Name of the investigators/co-investigators with designation & department**:

Pavan kumar Rudrabhatla

Junior resident

Department of Medicine

Guide: **Dr. Deepanjali S**

Assistant Professor

Department of Medicine

Co-guides  **Professor R P Swaminathan**

Senior Professor and HOD

Department of Medicine

**Dr. Jharna Mandal**

Associate Professor

Department of Microbiology

- 1. **Number of projects already with the investigators/co-investigators**:

Pavan kumar Rudrabhatla : 0

Dr. Deepanjali S : 0

Professor R.P.Swaminathan : 2

Dr. Jharna Mandal : 2

- 1. **Date of approval by JIPMER Research Council:** 20/8/2014
  2. **Sources of funding**: Not applicable
  3. **Objectives of the study:** To compare the clinical and microbiological efficacy of a shortened 7-day antibacterial strategy with the conventional 14-day strategy in hospitalized patients with non-severe acute pyelonephritis.
  4. **Justification for the conduct of the study:** Treatment of urinary tract infections which was once an easily treatable infection has become complicated in the era of widespread antibiotic resistance. However the conventional norm in the treatment of pyelonephritis has been 14 days.^1^ Recent studies which have directly compared shorter versus longer duration of treatment in acute pyelonephritis have been done in settings were uropathogens are still sensitive to fluoroquinolones.

A study published in JAMA in 2000 compared the efficacy of 7 day ciprofloxacin regimen to 14 days trimethoprim-sulfamethoxazole regimen in women with acute pyelonephritis. This randomized controlled trial found that the 7 day ciprofloxacin regimen was associated with greater bacteriological and clinical cure rates .^2^

A study done by Sandberg et al published in Lancet 2012, compared the efficacy of 7 days vs. 14 days treatment with ciprofloxacin in women with acute uncomplicated pyelonephritis. This study showed that the shorter regimen is successful even in older women.^3^

Recently a meta-analysis was done including 8 RCTs to answer the question whether 7 days or less treatment is non-inferior to longer regimens in acute pyelonephritis and septic uti.^4^  This systematic review found that even in bacteremic patients shorter treatment regimens are equivalent to longer ones in terms of bacteriological and clinical failure rates. However most of the RCTs included in this study are fluoroquinolone-based regimens. Authors of the meta-analysis emphasized the need for more studies using regimens based on other groups of antibiotics.

India is one amongst the five major consumers of antibiotics according to a study done based on national pharmaceutical sales data. ^5^ Alarming rates of antibiotic resistance amongst uropathogens has been noted in the country.^6 ‘^The Chennai declaration’, formulated in a joint meeting of medical societies of India, identified that increasing antimicrobial resistance is a serious global and regional challenge and there is an urgent need to initiate measures to tackle the scenario and join international efforts to control this menace.^7^

Shortening antibacterial regimens will help to decrease chances of spread of antimicrobial resistance and collateral damage. Also it cuts down duration of hospital stay, adverse effects and treatment costs.

**References:**

1. Gupta K, Hooton TM, Naber KG, Wullt B, Colgan R, Miller LG, et al. International Clinical Practice Guidelines for the Treatment of Acute Uncomplicated Cystitis and Pyelonephritis in Women: A 2010 Update by the Infectious Diseases Society of America and the European Society for Microbiology and Infectious Diseases. Clin Infect Dis. 2011 Mar 1;52(5):e103-20.
2. Talan DA, Stamm WE, Hooton TM, Morgan GJ, Burke T, Iravani A,et al. JAMA 2000 Mar 22-29;283(12):1583-90.
3. Sandberg T, Skoog G, Hermansson AB, Kahlmeter G, Kuylenstierna N, Lannergaard A, et al. Ciprofloxacin for 7 days versus 14 days in women with acute pyelonephritis: a randomized open-label and double-blind, placebo-controlled, non-inferiority trial. The Lancet. 2012 Aug 4 ;380(9840):484–90.
4. Eliakim-Raz N, Yahav D, Paul M, Leibovici L. Duration of antibiotic treatment for acute pyelonephritis and septic urinary tract infection-- 7 days or less versus longer treatment: systematic review and meta-analysis of randomized controlled trials. J Antimicrob Chemother. 2013 Oct;68(10):2183-91.
5. Van Boeckel TP, Gandra S, Ashok A, Caudron Q, Grenfell BT, Levin SA, et al. Global antibiotic consumption 2000 to 2010: an analysis of national pharmaceutical sales data. Lancet Infect Dis. 2014 Aug;14(8):742-50.
6. Mandal J, Acharya NS, Buddhapriya D, Parija SC. Antibiotic resistance pattern among common bacterial uropathogens with a special reference to ciprofloxacin resistant Escherichia coli. Indian J Med Res. 2012 Nov;136(5):842-9.
7. Ghafur A, Mathai D, Muruganathan A, Jayalal J, Kant R, Chaudhary D, et al. The Chennai declaration: A roadmap to tackle the challenge of antimicrobial resistance. Indian J Cancer. 2013 Jan-Mar;50(1):71-3.
   1. **Methodology: It should provide details of number of patients, inclusion criteria, exclusion criteria, control(s), study design, dosages of drug, duration of treatment, investigations to be done etc:**
8. **Study design:** Randomized controlled trial, two-arm, parallel group, open label, non-inferiority design
9. **Study participants**

- **Inclusion criteria**

Either sex aged >18 years, admitted for acute pyelonephritis, defined as fever of at least 38 ^o^C and at least one symptom or sign relating to the urinary tract such as flank pain, costovertebral angle tenderness, dysuria, urgency or frequency along with the microbiologic criteria (≥10 pus cells/hpf or dipstick leucocyte esterase test positive and a positive culture with ≥10^5^ CFU/ mL of urine). They should have improved with empirical or culture-guided antibiotic treatment and be afebrile for at least 48 hours by day 7 of antibiotic treatment.

- **Exclusion criteria**
- Catheter-associated UTI
- Recurrent UTI
- Obstructive uropathy
- Residual bladder urine >100 ml
- Acute or chronic prostatitis / prostatic abscess
- Emphysematous PN/renal or perinephric abscess
- Shock requiring vasopressors
- Altered sensorium requiring intubation (GCS score <8)
- Acute lung injury requiring mechanical ventilation
- Renal failure requiring RRT
- Clinically manifest DIC
- Pregnancy
- Any immunosuppressive treatment
- **Number of groups to be studied, identify groups with definition**: 2

CONTROLS: Patients randomized to 14 days treatment arm

INTERVENTION: Patients randomized to 7 days treatment arm

1. **Sampling**

- **Sampling population**

Patients admitted to medical or emergency wards of JIPMER with non-severe acute pyelonephritis.

- **Sampling size calculation**

Assuming efficacy of 14-day treatment with standard intravenous agents to be 95%, to show that the upper limit of the difference in retreatment rates does not exceed 15%, with 80% power and one-sided alpha 5%, 27 patients are required in each arm. To compensate for 15% loss to follow up, 31 patients are required in each arm. A 15% non-inferiority margin translates to 33% decrease in antibiotic use.

- **Sampling technique**

Consecutive eligible patients admitted during the study period will be randomized.

1. **Randomization details (for interventional studies)- intervention details with standardization techniques (drugs/ devices/ invasive procedures/ noninvasive procedures** **others)**

The study plans to randomize non severe acute pyelonephritis patients who have improved on antibiotics and are afebrile for at least 48 hours on the Day 7 of appropriate antibiotic treatment into control and intervention arms. Since the sample size is comparatively small, to maintain balance between the intervention and control groups for the main prognostic variables, a method of minimization will be used for treatment allocation.

The factors to be minimized are

- Presence/absence diabetes mellitus
- Presence/absence acute kidney injury (peak creatinine >2 mg/dL)
- Based on whether the patient is on empirical/revised regimen
- Whether treatment with aminoglycoside based regimen
- sex
- age > 55 years

Details of consecutive patients are provided to a software program which assigns the patients to either group. To maintain allocation concealment a biased coin technique will be used with a pre-specified cut-off. Thus the process will not be completely deterministic.

1. **Study procedure**

The study will be done in the medical and emergency wards in JIPMER. Consecutive patients who are admitted as suspected cases of pyelonephritis will be screened to assess eligibility for the trial. On the day they are admitted, a thorough history of the disease with all the relevant past illnesses and treatment details will be taken aided by a standard proforma. A physical examination will be done. If the clinical suspicion for acute pyelonephritis is high then urine microscopic examination for pus cells or casts or urine dipstick test for leucocyte esterase will be done. Along with this a urine culture sample will be sent before the first dose of antibiotic is administered. Also blood will be drawn for blood culture, hemogram, renal function tests as well as liver function tests. An USG KUB will be done. Patients will be started on an empirical antibiotic regimen as decided by the treating physician. Subsequently daily clinical assessment will be done specifically noting any clinical improvement, worsening or appearance of new symptoms. For patients who do not improve on an empirical regimen a culture-guided revised regimen will be started as per the treating physicians' discretion. At Day 4, a repeat urine culture will be sent. If this culture is sterile and the patient consistently maintains clinical improvement by Day 7, with no fever recorded for at least 48 hours, patients will be randomized to either one of the arms after obtaining informed written consent. Throughout this procedure previously mentioned criteria will be used to exclude patients from the trial, if warranted. Patients randomized to treatment arm are discharged after completion of 7 days of antibiotics while those randomized to control arm are given further 7 days of same antibiotic.

The follow-up clinical assessments are done 1week post-discharge and 6 weeks post-treatment. Repeat urine cultures are also performed during follow-up visits to assess microbiologic cure rates. Patients presenting with relapse or re infection (within the follow up period) would be treated for 14 days of antibiotic therapy. The following algorithm depicts the process for better understanding.

**ALGORITHM** Patients admitted with features of acute pyelonephritis

On Day 0 urine microscopy/dipstick test, blood counts

baseline urine C&S, blood C&S, RFT, LFT, USG KUB

Empirical antibiotic(s) decided by treating physician

Daily clinical assessment for symptomatic improvement

Baseline urine culture report collected on Day 3

Culture negative

Not eligible

Organism sensitive to empirical regimen Organism not sensitive to empirical regimen

& Clinical improvement present or no cinical improvement

Revised regimen

Send day 4 urine culture Send urine culture on day 4 of revised regimen

Culture positive Culture positive

Exclude Exclude

Culture negative & continued

improvement on day 7

INFORMED WRITTEN CONSENT

RANDOMISE

7 days arm 14 days arm

Discharge Continue same regimen for 7 more days

Follow up clinical assessment and urine cultures 1 week post discharge and 6 weeks post treatment

- 1. **Permission from Drug Controller General of India (DCGI) if applicable:**  Not applicable
  2. **Costs involved (Appx. in Rs.)** : Not applicable
  3. **Investigations:** A physical examination of the patient will be done. If the clinical suspicion for acute pyelonephritis is high then urine microscopic examination for pus cells or casts or urine dipstick test for leucocyte esterase will be done. Along with this a urine culture sample will be sent before the first dose of antibiotic is administered. Also blood will be drawn for blood culture, hemogram, renal function tests as well as liver function tests. An USG KUB will be done.

Urine culture will be sent again on

- Day 4 of sensitive empirical treatment or day 4 of revised regimen.
- 1 week post discharge
- 6 weeks post treatment
  1. **Who will bear the costs of the requirements?** Not applicable

a. Patient b Project c. Exempt d. Other Agencies (Name)

[Type a quote from the document or the summary of an interesting point. You can position the text box anywhere in the document. Use the Text Box Tools tab to change the formatting of the pull quote text box.]

[Type a quote from the document or the summary of an interesting point. You can position the text box anywhere in the document. Use the Text Box Tools tab to change the formatting of the pull quote text box.]

[Type a quote from the document or the summary of an interesting point. You can position the text box anywhere in the document. Use the Text Box Tools tab to change the formatting of the pull quote text box.]

- 1. **Ethical issues involved in the study: (***less than minimal risk / minimal risk / more than minimal risk to the study subjects (for guidance please consult ICMR guidelines - at JIPMER website)*  [Along with the level of risk, the risks should be written in detail. If you feel there will be

no risk, give justification]

There is minimal risk involved. In case the 7 day strategy is actually inferior to the 14 day strategy, there will be higher chances of re-treatment amongst the patients assigned to treatment arm.

- 1. **Do you need exemption from obtaining Informed Consent from study subjects - if so give justifications:** Not applicable
  2. **Whether Consent forms part 1 and 2 in English and in local language are enclosed?**

*(if the consent form in local language is not applicable, appropriate explanations must*

*be provided) : Yes*

- 1. **Documents attached**
  2. Brief CV of investigators (including no. of projects with him/her) - Needed only for Investigator/s from outside JIPMER
  3. Investigator’s Brochure
  4. Others
  5. **Conflict of interest for any other investigator(s) (if yes, please explain in brief)** : No
  6. **Whether mandatory soft copy of the proforma (CD) has been attached?** : Yes
  7. **We, the undersigned, have read and understood this protocol and hereby agree to conduct the study in accordance with this protocol and to comply with all requirements of the ICMR guidelines (2006)**

Signature of the Investigators: Date:

Signature of the Head of the Department Date:

**JAWAHARLAL INSTITUTE OF POSTGRADUATE MEDICAL EDUCATION & RESEARCH, PUDUCHERRY-605006**

(Institute of national importance under Govt. of India)

**Format**

FOR SUBMITTING PG DISSERTATION PROPOSAL FOR CONSIDERATION BY PG RESEARCH MONITORING COMMITTEE

**PART A-GENERAL INFORMATION**

1. Title of the dissertation: Comparison of 7 days versus 14 days antibacterial treatment for hospitalized non severe acute pyelonephritis patients – a randomized controlled trial
2. Name of the candidate with : Pavan kumar Rudrabhatla

mobile numbers, email ID Mob : 9159525608

email id : [pavankumarrudrabhatla@gmail.com](mailto:pavankumarrudrabhatla@gmail.com)

1. Name of the course studying : Junior Resident

Department of Medicine

1. Year of admission : 2014
2. Month and year of appearing : March 2017

for final examination

1. Month and year of submitting : August 2016

dissertation

1. Name(s), designation(s) & addresses : **Dr. Deepanjali S**

Assistant Professor

Department of Medicine

Mob : 9488819981

email id : [deepanjali.s@jipmer.edu.in](mailto:deepanjali.s@jipmer.edu.in)

**Professor R P Swaminathan**

Senior Professor and HOD

Department of Medicine

Mob : 9500941171

email id : [rpsmed@gmail.com](mailto:rpsmed@gmail.com)

**Dr. Jharna Mandal**

Associate Professor

Department of Microbiology

Mob: 9677451239

email id : jharna.m@jipmer.edu.in

1. A. State whether it is : Interdepartmental

intradepartmental or interdepartmental

B. If the study is interdepartmental

1. State the names of collaborating departments : Microbiology
2. State whether consent has been obtained from them : Yes
3. Total funds required for the study(in rupees) : Not applicable
4. Source of funding : Not applicable

**PART B-TECHNICAL DETAILS**

1. **Title of the dissertation**: “Comparison of 7 days versus 14 days antibacterial treatment for hospitalized non severe acute pyelonephritis patients – a randomized controlled trial”
2. **Introduction**

**A. Problem statement**

Urinary tract infections are one of the most common diseases for which antibacterials are used in community as well as hospital settings. Widespread antibacterial resistance amongst uropathogens in the Asia-Pacific regions has limited the use of oral antibacterials for acute pyelonephritis. Hence treatment with intravenous antibacterials has become the norm. While studies done using oral fluoroquinolones (with or without initial intravenous agents) have shown that treatment of acute uncomplicated pyelonephritis may be limited to 5-7 days, such data is not available for other commonly used intravenous antibacterial agents. Moreover, there is no data available about duration of treatment with non fluoroquinolone antibacterials in less severe cases of complicated acute pyelonephritis.

**B. Rationale**

Patients with acute pyelonephritis are hospitalized either because of severity of disease or more commonly because only intravenous antibacterials are the only option available. A recent meta-analysis has shown that even in septic UTI 7 days of antibacterial treatment is sufficient. However no recent trials are available which have done head to head comparison between shorter versus longer regimens of non-fluoroquinolone intravenous antibacterials like cephalosporins, carbapenems or aminoglycosides. Shortening antibacterial regimens will help to decrease chances of spread of antimicrobial resistance and collateral damage. Also it cuts down duration of hospital stay, adverse effects and treatment costs.

**C. Novelty**

Recent trials about optimal duration of antibacterials in acute pyelonephritis are on fluoroquinolones-based regimens. The present study addresses the question of duration of treatment with non-fluoroquinolone based regimens and attempts to answer the question whether a shorter regimen is non-inferior to conventional 14 days treatment.

**D. Expected outcome and application**

If the shorter treatment regimen is proven non-inferior to the longer one, it will directly translate to an evidence base for the routine clinical care of acute pyelonephritis patients. Patients will benefit in terms of shorter hospital stay and lesser chances for adverse effects from antibacterial use. It will also be a positive step towards antibiotic stewardship.

**3. Research question(s)**

Is 7 days antibacterial treatment with sensitive antibacterial/antibacterials for hospitalized non severe acute pyelonephritis patients is non- inferior to 14 days antibacterial treatment?

1. **Research hypothesis (s), if any:** “Antibacterial treatment with sensitive antibacterial/antibacterials for 7 days is *non-inferior* to 14-day treatment in hospitalised non severe acute pyelonephritis patients.”
2. **Aims and objectives**

**Primary objective:** To compare the clinical and microbiological efficacy of a shortened 7-day antibacterial strategy with the conventional 14-day strategy in hospitalised patients with non severe acute pyelonephritis

1. **Review of literature**

Treatment of urinary tract infections which was once an easily treatable infection has become complicated in the era of widespread antibiotic resistance. Two professional societies have issued guidelines for treatment of acute pyelonephritis. The IDSA guidelines are mainly for acute uncomplicated pyelonephritis and recommends shortened fluoroquinolone-based regimens.^1^ According to the European Association of Urology the duration of treatment for complicated cases of pyelonephritis ranges from 1-4 weeks.^2^ However the conventional norm in the treatment of pyelonephritis has been 14 days.^3^ Recent studies which have directly compared the duration of treatment in acute pyelonephritis have been done in settings were uropathogens are still sensitive to fluoroquinolones.

A study published in JAMA in 2000 compared the efficacy of 7 day ciprofloxacin regimen to 14 days trimethoprim-sulfamethoxazole regimen in women with acute pyelonephritis. This randomized controlled trial found that the 7 day ciprofloxacin regimen was associated with greater bacteriological and clinical cure rates .^4^

A study done by Sandberg et al published in Lancet 2012, compared the efficacy of 7 days vs. 14 days treatment with ciprofloxacin in women with acute uncomplicated pyelonephritis. This study showed that the shorter regimen is successful even in older women.^5^

Recently a meta-analysis was done including 8 RCTs to answer the question whether 7 days or less treatment is non-inferior to longer regimens in acute pyelonephritis and septic uti.^6^ This systematic review found that even in bacteremic patients shorter treatment regimens are equivalent to longer ones in terms of bacteriological and clinical failure rates. However most of the RCTs included in this study are FQ-based regimens. One of the RCTs which included ceftriaxone as an intravenous agent for treatment in pyelonephritis used it only for initial days to be followed by oral cefixime.^7^ The other two RCTs which included beta-lactam agents were done before the 1990s.^8,9^ Authors of the meta-analysis emphasized the need for more studies using regimens based on other groups of antibiotics.

Symposium held at Chennai in 2012 ‘ the Chennai declaration’ suggested proper dosage and duration of antibiotics is helpful in reducing antibiotic resistance. ^10^

India is one amongst the five major consumers of antibiotics according to a study done based on national pharmaceutical sales data. ^11^  Alarming rates of antibiotic resistance amongst uropathogens has been noted in the country. ^12,13,14^ A guidelines for treatment of complicated uti in Asia-pacific region was proposed recently.^15^ It is noteworthy that only intravenous antibacterial options are available in the face of rampant antimicrobial resistance. However the optimal duration of antibacterial treatment finds no specific mention in these guidelines.

1. **Methodology**

**Study design:** Randomized controlled trial, two-arm, parallel group, open label, non-inferiority design

**Study participants**

- **Inclusion criteria**

Either sex aged >18 years, admitted for acute pyelonephritis, defined as fever of at least 38^o^C and at least one symptom or sign relating to the urinary tract such as flank pain, costovertebral angle tenderness, dysuria, urgency or frequency along with the microbiologic criteria (≥10 pus cells/hpf or dipstick leucocyte esterase test positive and a positive culture with ≥10^5^ CFU/ mL of urine). They should have improved with empirical or culture-guided antibiotic treatment and be afebrile for at least 48 hours by day 7 of antibiotic treatment.

- **Exclusion criteria**
- Catheter-associated UTI
- Recurrent UTI
- Obstructive uropathy
- Residual bladder urine >100 ml
- Acute or chronic prostatitis / prostatic abscess
- Emphysematous PN/renal or perinephric abscess
- Shock requiring vasopressors
- Altered sensorium requiring intubation (GCS score <8)
- Acute lung injury requiring mechanical ventilation
- Renal failure requiring RRT
- Clinically manifest DIC
- Pregnancy
- Any immunosuppressive treatment
- **Number of groups to be studied, identify groups with definition**: 2

CONTROLS: Patients randomized to 14 days treatment arm

CASES : Patients randomized to 7 days treatment arm

**Sampling**

- **Sampling population**: Patients admitted to medical or emergency wards of JIPMER with non severe acute pyelonephritis.
- **Sampling size calculation:** Assuming efficacy of 14-day treatment with standard intravenous agents to be 95%, to show that the upper limit of the difference in retreatment rates does not exceed 15% with 80% power and one-sided alpha 5% 27 patients are required in each arm. To compensate for 15% loss to follow up, 31 patients are required in each arm. A 15% non-inferiority margin translates to 33% decrease in antibiotic use.
- **Sampling technique**

Consecutive eligible patients admitted during the study period will be randomized.

**Randomization details (for interventional studies)- intervention details with standardization techniques (drugs/ devices/ invasive procedures/ noninvasive procedures** **others)**

The study plans to randomize non severe acute pyelonephritis patients who have improved on antibiotics and are afebrile for at least 48 hours on the Day 7 of appropriate antibiotic treatment into control and intervention arms. Since the sample size is comparatively small, to maintain balance between the intervention and control groups for the main prognostic variables, a method of minimization will be used for treatment allocation. The factors to be minimized are

- Presence/absence diabetes mellitus
- Presence/absence acute kidney injury (peak creatinine >2 mg/dL)
- Based on empirical/revised regimen
- Treatment with aminoglycoside based regimen
- sex
- age > 55 years

Details of consecutive patients are provided to a software program which assigns the patients to either group. To maintain allocation concealment a biased coin technique will be used with a pre-specified cut-off. Thus the process will not be completely deterministic.

**Study procedure**

The study will be done in the medical and emergency wards in JIPMER. Consecutive patients who are admitted as suspected cases of pyelonephritis will be screened to assess eligibility for the trial. On the day they are admitted, a thorough history of the disease with all the relevant past illnesses and treatment details will be taken aided by a standard proforma. A physical examination will be done. If the clinical suspicion for acute pyelonephritis is high then urine microscopic examination for pus cells or casts or urine dipstick test for leucocyte esterase will be done. Along with this a urine culture sample will be sent before the first dose of antibiotic is administered. Also blood will be drawn for blood culture, hemogram, renal function tests as well as liver function tests. An USG KUB will be done. Patients will be started on an empirical antibiotic regimen as decided by the treating physician. Subsequently daily clinical assessment will be done specifically noting any clinical improvement, worsening or appearance of new symptoms. For patients who do not improve on an empirical regimen a culture-guided revised regimen will be started as per the treating physicians' discretion. At Day 4 a repeat urine culture will be send. If this culture is sterile and the patients consistently maintains clinical improvement by Day 7, with no fever recorded for at least 48 hours, patients will be randomized to either one of the arms after obtaining a informed written consent. Throughout this procedure previously mentioned criteria will be used to exclude patients from the trial, if warranted. Patients randomized to treatment arm are discharged after completion of 7 days of antibiotics while those randomized to control arm are given further 7 days of same antibiotic. The follow-up clinical assessments are done 1week post-discharge and 6 weeks post-treatment. Repeat urine cultures are also performed during follow-up visits to assess microbiologic cure rates. Patients presenting with relapse or re infection (within the follow up period) would be treated for 14 days of antibiotic therapy.

1. **Data collection methods including settings and periodicity**

Data will be collected by direct interview with patients aided by a proforma. Physical examinations are repeated for daily clinical assessment. The temperature charts as well as treatment charts of patients will be reviewed. Data on laboratory and radiological parameters will be recorded. Patients are clinically assessed daily until they are discharged and then re-assessed on subsequent two follow-up visits on opd basis. If re-treatment is required those patients will be followed up in wards.

1. **If the clinical trial, whether registration with CTRI will be done**:

Yes

1. **Are the drugs/ devices to be used approved for these indications by drug controller general of India (DCG-1) ? (Enclose the approval letter for the drug/device from DCG-1** **for trial on humans)**

Not applicable

1. **List of variables and their measurement methods with standardization techniques**
2. **Independent variables**

Allotment of patients into 7 days and 14 days treatment arms.

1. **Outcome variables**
2. Re-treatment rates within 6 weeks

(due to clinical or microbiological failure; blinded adjudication)

1. Duration of hospital stay
2. Daily defined dose of antibiotics
3. Asymptomatic bacteriuria
4. Adverse effects of antibiotics
5. **Confounding and interacting variables**

- Presence/absence diabetes mellitus
- Presence/absence acute kidney injury (peak creatinine >2 mg/dL)
- Based on empirical/revised regimen
- Treatment with aminoglycoside based regimen
- sex
- age > 55 years

1. **List variable wise statistical tests to be used for data analysis**

Duration of hospital stay and daily defined dose of antibiotics would be analyzed by Wilcoxon rank sum test. Asymptomatic bacteriuria and adverse affects would be analysed by Fisher’s exact test.

1. **List risks and benefits of the study**

**Risks:** In case the 7 day strategy is actually inferior to the 14 day strategy, there will be higher chances of re-treatment amongst the patients assigned to treatment arm.

**Benefits:** Patients assigned to 7 day arm will receive lesser total amount of antibacterials, so lesser chances for side-effects and lesser duration of hospital stay and risk of nosocomial infections. If the shorter regimen is found to be non-inferior, then the significant decrease in antibiotic use will help to cut down treatment costs and will help prevent development as well as spread of antibiotic resistance.

1. **Relevant references for the project**

(Minimum 10, Maximum 20) (in Vancouver style)]

1. Gupta K, Hooton TM, Naber KG, Wullt B, Colgan R, Miller LG, et al. International Clinical Practice Guidelines for the Treatment of Acute Uncomplicated Cystitis and Pyelonephritis in Women: A 2010 Update by the Infectious Diseases Society of America and the European Society for Microbiology and Infectious Diseases. Clinical Infectious Diseases. 2011 Mar 1;52(5):e103–e120.
2. Warren JW, Abrutyn E, Hebel JR, Johnson JR, Schaeffer AJ, Stamm WE.Clin Infect Dis. 1999 Oct;29(4):745-58.
3. Clinical approach to initial choice of antimicrobial therapy.In: Gilbert DV, Moellering RC, Eliopoulos GM, Chambers HF, Saag MS (eds.) The Sanford Guide To Antimicrobial Therapy *. 43rd* ed.USA: Antimicrobial therapy, Inc; 2013. P34 - 35.
4. Talan DA, Stamm WE, Hooton TM,Morgan GJ, Burke T, Iravani A, Reuning-Scherer J, Church DA. JAMA. 2000 Mar 22-29;283(12):1583-90.
5. Sandberg T, Skoog G, Hermansson AB, Kahlmeter G, Kuylenstierna N, Lannerg\a ard A, et al. Ciprofloxacin for 7 days versus 14 days in women with acute pyelonephritis: a randomised, open-label and double-blind, placebo-controlled, non-inferiority trial. The Lancet. 2012;380(9840):484–90.
6. Eliakim-Raz N, Yahav D, Paul M, Leibovici L. Duration of antibiotic treatment for acute pyelonephritis and septic urinary tract infection-- 7 days or less versus longer treatment: systematic review and meta-analysis of randomized controlled trials. Journal of Antimicrobial Chemotherapy [Internet]. 2013 May 21 [cited 2014 Jul 28]; Available from: http://www.jac.oxfordjournals.org/cgi/doi/10.1093/jac/dkt177
7. Monmaturapoj T, Montakantikul P, Mootsikapun P, Tragulpiankit P. A prospective, randomized, double dummy, placebo-controlled trial of oral cefditoren pivoxil 400mg once daily as switch therapy after intravenous ceftriaxone in the treatment of acute pyelonephritis. International Journal of Infectious Diseases. 2012 Dec;16(12):e843–e849.
8. Jernelius H, Zbornik J, Bauer CA. One or three weeks’ treatment of acute pyelonephritis? A double-blind comparison, using a fixed combination of pivampicillin plus pivmecillinam. Acta Med Scand 1988; 223: 469–77.
9. OdeB, Bro¨msM,WalderMet al. Failure of excessive doses of ampicillin to prevent bacterial relapse in the treatment of acute pyelonephritis. Acta Med Scand 1980; 207: 305–7.
10. Ghafur A, Mathai D, Muruganathan A, Jayalal J, Kant R, Chaudhary D, et al. The Chennai declaration: A roadmap to tackle the challenge of antimicrobial resistance. Indian Journal of Cancer. 2013;50(1):71.
11. Van Boeckel TP, Gandra S, Ashok A, Caudron Q, Grenfell BT, Levin SA, et al. Global antibiotic consumption 2000 to 2010: an analysis of national pharmaceutical sales data. The Lancet infectious diseases [Internet]. 2014 [cited 2014 Jul 31]; Available from: http://www.sciencedirect.com/science/article/pii/S1473309914707807
12. Mandal J, Acharya NS, Buddhapriya D, Parija SC. Indian J Med Res 136, Nov 2012:842-49.
13. Mukherjee M. Multidrug-Resistance and Extended Spectrum Beta-Lactamase Production in Uropathogenic E. Coli which were Isolated from Hospitalized Patients in Kolkata, India. JOURNAL of CLINICAL AND DIAGNOSTIC RESEARCH [Internet]. 2013 [cited 2014 Jul 28]; Available from: http://www.jcdr.net/article_fulltext.asp?issn=0973-709x&year=2013&month=March&volume=7&issue=3&page=449-453&id=2796
14. Kothari A, Sagar V. Antibiotic resistance in pathogens causing community-acquired urinary tract infections in India: a multicenter study. J Infect Dev Ctries 2008;
15. Hsueh P-R, Hoban DJ, Carmeli Y, Chen S-Y, Desikan S, Alejandria M, et al. Consensus review of the epidemiology and appropriate antimicrobial therapy of complicated urinary tract infections in Asia-Pacific region. Journal of Infection. 2011 Aug;63(2):114–23.
